# Association of the intermediate filament nestin with cancer stage: a meta-analysis based on 223 positive/high nestin cases and 460 negative/low case-free controls

**DOI:** 10.18632/oncotarget.4042

**Published:** 2015-05-08

**Authors:** Beilong Zhong, Tao Wang, Jianyong Zou, Fangfang Zheng, Rijiao Huang, Xiaobin Zheng, Weilin Yang, Zhenguang Chen

**Affiliations:** ^1^ Department of Thoracic Surgery, The Fifth Affiliated Hospital, Sun Yat-sen University, Zhuhai, Guangdong, China; ^2^ Center for Stem Cell Biology and Tissue Engineering, Sun Yat-sen University, Key Laboratory for Stem Cells and Tissue Engineering, Ministry of Education, Guangzhou, Guangdong, China; ^3^ Department of Biochemistry, Zhongshan Medical School, Sun Yat-sen University, Guangzhou, Guangdong, China; ^4^ Department of Thoracic Surgery, The First Affiliated Hospital, Sun Yat-sen University, Guangzhou, Guangdong, China; ^5^ Lung Cancer Research Center of Sun Yat-sen University, Guangzhou, Guangdong, China; ^6^ Department of Pediatrics, The Fifth Affiliated Hospital, Sun Yat-sen University, Zhuhai, Guangdong, China; ^7^ Department of Clinical Laboratory, The Fifth Affiliated Hospital, Sun Yat-sen University, Zhuhai, Guangdong, China; ^8^ Department of Respiratory Medicine, The Fifth Affiliated Hospital, Sun Yat-sen University, Zhuhai, Guangdong, China; ^9^ Department of Cardiothoracic Surgery of East Division, The First Affiliated Hospital, Sun Yat-sen University, Guangzhou, Guangdong, China

**Keywords:** nestin, intermediate filament, cancer stage, TNM staging, meta-analysis

## Abstract

**Background:**

Nestin, a member of the intermediate filament protein family, has been reported to be associated with several types of neoplastic transformation. However, questions remain, with studies reporting sometimes inconclusive or conflicting data. Thus, the aim of this study was to evaluate literature reports on the relationship between nestin and cancer stage.

**Methods:**

Relevant articles published as of June 2014 were retrieved from multiple databases. After applying specific inclusion criteria, we chose seven articles relating to nestin expression and cancer stage, which included a total of 223 positive/high nestin cases and 460 negative/low case-free controls.

**Results:**

Overall, positive/high nestin was significantly associated with median or advanced stages of several types of cancer (nestin and cancer stage: OR = 1.90, 95% CI = 1.30–2.78; nestin and lymph node: OR = 2.17, 95% CI = 1.26–3.72). Notably, studies relating to lung cancer (three qualifying articles) showed a significant association between nestin and lung cancer stage (OR = 2.00, 95% CI = 1.16–3.44).

**Conclusion:**

These findings indicate that positive/high nestin may be more strongly linked to median or advanced cancer stage and correlated with malignant characteristics that lead to poor prognosis in different cancers, especially lung cancer.

## INTRODUCTION

Nestin, a member of the class VI intermediate filament protein family, is expressed in normal cells of different tissues and organs.[3] Nestin expression was first reported in progenitor cells of central and peripheral nervous systems.[4] Its expression has since been reported in skeletal and cardiac muscle progenitors, developing tooth buds, testis and hair follicle sheath progenitor cells of the skin, a distribution that suggests that nestin is a potential indicator of proliferative and multipotency status in several types of progenitor cells.[5, 6-11]

Recent reports have indicated that nestin might be linked to malignant characteristics, suggesting that abundant nestin expression correlates with greater malignancy and poorer prognosis in different cancers. Moreover, several studies have also reported that nestin is a marker of cancer stem cells in different malignancies, including brain tumors, uterine cancer, cervical cancer, prostate cancer, bladder cancer, head and neck cancer, ovarian cancer, testicular cancer, pancreatic cancer, and malignant rhabdoid tumors.[12-19]

The TNM (primary Tumor, lymph Nodes, and distant Metastasis) classiﬁcation system, published by the American Joint Committee on Cancer (AJCC) and the Union for International Cancer Control (UICC) for Malignant Tumors, has been used worldwide and across all medical specialties as a cancer-staging technique.[1] The TNM system is based on the anatomic extent of the tumor, as determined clinically and, in most instances, histopathologically, and can facilitate diagnosis, prognosis, treatment and other clinical decisions. [1, 2]

Although several articles on the relationship between nestin and cancer stage have been reported, including Masao *et al.*,[20] Shinichiro *et al.*,[21] Chen *et al.*[22-23] and Karsten *et al.*[24], only that of Karsten *et al.* reported that high nestin was strongly, and significantly, associated with cancer stage. Because of the relatively small sample sizes and limited statistical power of these individual, inconclusive studies, it is important to summarize the results from different studies to more accurately assess the association of positive/high nestin with cancer stage. To this end, we carried out a meta-analysis of all eligible studies, combining results to produce a single estimate of the major effect with enhanced precision.[25] We further performed a subgroup analysis stratified according to lymph node involvement and cancer category.

## RESULTS

### Study characteristics

**Figure 1:**
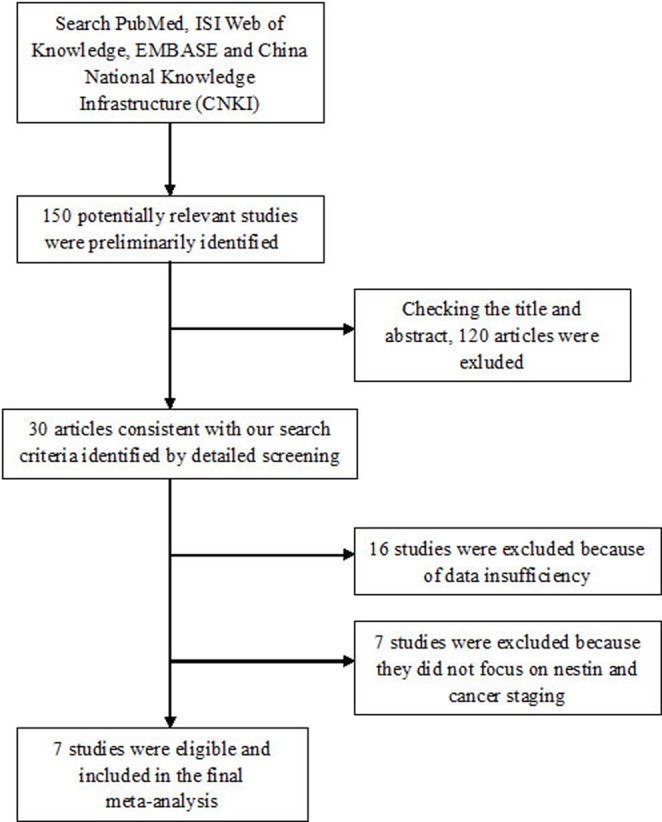
Flow chart of study selection

Our search strategy identified 150 potentially relevant studies. After checking the title and abstract, a total of 30 articles consistent with our search criteria were preliminarily chosen for further detailed evaluation. After careful screening, we excluded 16 studies because data were insufficient for analysis. Of the remaining 14 studies, seven did not focus on nestin and cancer staging. Ultimately, seven studies on nestin and cancer staging that included a total of 223 positive/high nestin cases and 460 negative/low case-free controls were deemed eligible for the final analysis. The characteristics of the included studies are listed in Table 1. All studies were related to clinical research, including three lung cancer studies, one pancreatic cancer study, one prostate cancer study, one gastric adenocarcinoma study, and one oral cancer study. All cancers were confirmed pathologically. The study selection process is shown schematically in Figure 1.

**Table 1:** Characteristics of the studies included in the meta-analysis

First author	Year	Country	Ethnicity	Cancer type	Total No. of patients	Median age (years)
Masao	2009	Japan	Asian	PC	60	64.6
Karsten	2009	Norway	Germanic	Prostate cancer	104	62.0
Chen	2010	China	Asian	Lung cancer	52	58.2
Shinichiro	2010	Japan	Asian	Lung cancer	171	64
Sadhna	2011	USA	Caucasian	GA	159	63
Mascolo	2012	Italy	Germanic	Oral cancer	66	60.5
Chen	2014	China	Asian	Lung cancer	71	57.6

PC, pancreatic cancer; GA, gastric adenocarcinoma.

### Meta-analysis results

The frequency of tumors, lymph node involvement, and cancer stage in positive/high and negative/low nestin patients are shown in detail in Table 2. All TNM staging data were collected, and a subset of lymph node data was extracted.

**Figure 2:**
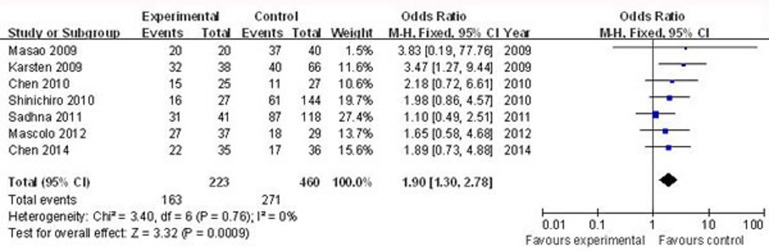
Forest plots of nestin and median/advanced cancer stage among all cases (positive/high nestin *vs*. negative/low nestin) The squares and horizontal lines correspond to the study-specific OR and 95% CI, respectively. The area of the squares reflects the weight (inverse of the variance). The diamond represents the summary OR and 95% CI.

Seven articles that included a total of 223 cases and 460 controls were used to evaluate the relationship between nestin with cancer stage. The main results of this meta-analysis are indicated in Figure 2. Overall, there was evidence for an association between positive/high nestin and median/advanced cancer stage in different cancers when all eligible studies were pooled into the meta-analysis. As show in Figure 2, the significant main effect observed was for nestin and median/advanced cancer stage (positive/high nestin *vs*. negative/low nestin: OR = 1.90, 95% CI = 1.30–2.78, *P* = 0.0009).

**Figure 3:**
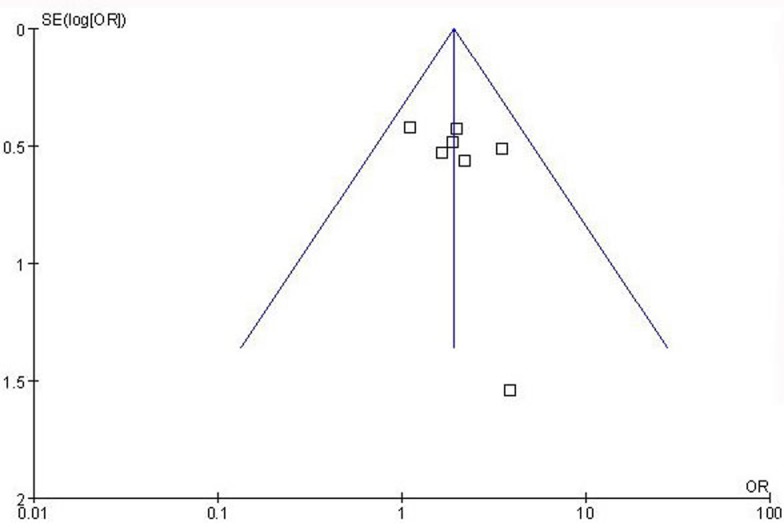
Funnel plot assessing evidence of publication bias from seven studies (median or advanced stage *vs*. early stage)

**Figure 4:**
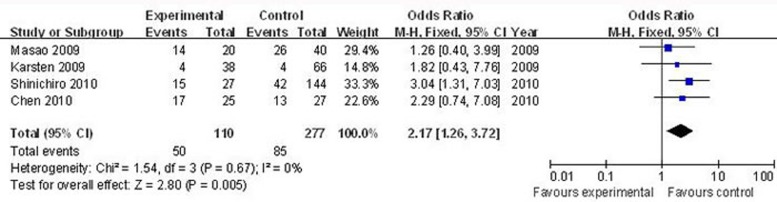
Forest plots of nestin and lymph node involvement among all cases (N_1-2_ *vs*. N_0_) The squares and horizontal lines correspond to the study-specific OR and 95% CI, respectively. The area of the squares reflects the weight (inverse of the variance). The diamond represents the summary OR and 95% CI.

**Figure 5:**
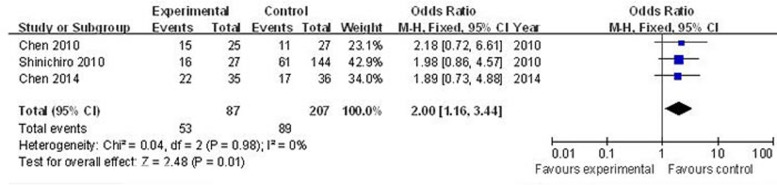
Forest plots of nestin and lung cancer stage in three cases (median or advanced stage *vs*. early stage) The squares and horizontal lines correspond to the study-specific OR and 95% CI, respectively. The area of the squares reflects the weight (inverse of the variance). The diamond represents the summary OR and 95% CI.

Four articles that included 110 cases and 277 controls were used to evaluate the relationship between nestin and lymph node involvement. In the analysis stratified by lymph node involvement (N_1-2_ *vs*. N_0_), as shown in Figure 4, the main effect remained (positive/high nestin *vs*. negative/low nestin: OR = 2.17, 95% CI = 1.26–3.72, *P* = 0.005). For the analysis stratified by cancer category, three articles that included 87 cases and 207 controls were used to evaluate the relationship between nestin and lung cancer (median or advanced stage *vs*. early stage). As show in Figure 5, the main effect remained in studies of lung cancer (positive/high nestin *vs*. negative/low nestin: OR = 2.00, 95% CI = 1.16–3.44, *P* = 0.01).

**Figure 6:**
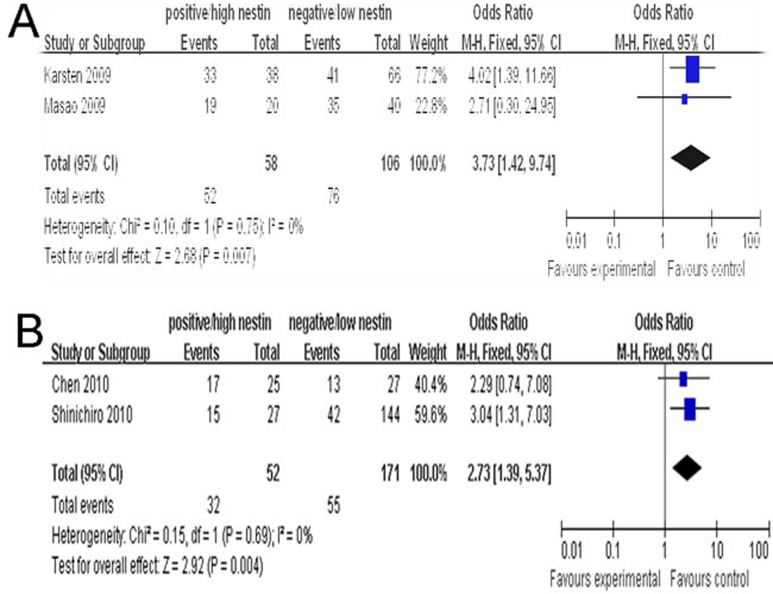
**A.** Forest plots of nestin and T staging of lung cancer in two cases (T_3-4_ *vs*. T_1-2_). **B.** Forest plots of nestin and N staging of lung cancer in two cases (N_1-2_ *vs*. N_0_). The squares and horizontal lines correspond to the study specific OR and 95% CI. The area of the squares reflects the weight (inverse of the variance). The diamond represents the summary OR and 95% CI.

More importantly, regarding to focus at the association of nestin with lung cancer staging, as shown in Figure 6, we have done some analysis on the relationship between nestin and T staging or N staging. In the analysis stratified by T staging (T_3-4_ *vs.* T_1-2_) in lung cancer, the main effect remained (positive/high nestin *vs*. negative/low nestin: OR = 3.73, 95% CI = 1.42–9.74, *P* = 0.007). Meanwhile, in the analysis stratified by N staging (N_1-2_ *vs*. N_0_) in lung cancer, the main effect remained (positive/high nestin *vs*. negative/low nestin: OR = 2.73, 95% CI = 1.39–5.37, *P* = 0.004).

### Tests of heterogeneity

Tests of heterogeneity between trials using the Q statistic showed no significant heterogeneity in the following analyses: nestin expression with cancer staging (positive/high nestin *vs*. negative/low nestin, *P* = 0.76, *I^2^* = 0%), nestin expression with lymph node positivity (positive/high nestin *vs*. negative/low nestin (*P* = 0.67, *I^2^* = 0%), and nestin expression with lung cancer stage, positive/high nestin *vs*. negative/low nestin (*P* = 0.98, *I^2^* = 0%). Accordingly, data were analyzed using a fixed-effects model.]

### Publication bias

Funnel plots were performed to assess publication bias. As show in Figure 3, the funnel plots revealed no obvious asymmetry at any cancer stage (median or advanced stage *vs*. early stage), and the results revealed no publication bias (*P* > 0.05).

## DISCUSSION

TNM stage is determined according to the outcomes of physical examination, biopsy, and imaging tests; in clinical application, a pathological report is the basis of TNM staging.[27] In addition to anatomic tumor categories, most sites and tumor types are classified according to TNM stage, with the anatomic extent of the tumor determining the cancer stage.[1, 2] Clearly distinguishing between stages and reliably estimating survival probability require that each stage be homogeneous. Stages I to III correspond to progressively advanced localized and regional cancer, whereas stage IV corresponds to systemic metastases. An accurate staging system is important for guiding physicians in treatment options and determining patients' prognosis.[28]

Nestin was initially discovered based on its expression in neural progenitor cells, where it was considered a marker for distinguishing precursor cells from differentiated cells.[29, 30] Subsequent reports have shown that nestin is expressed in breast, prostate and pancreatic cancer, and is positively correlated with tumor malignancy.[31-33] But some findings regarding nestin are inconclusive or controversial, especially in the context of cancer staging.

Although a number of studies investigating the association of nestin with cancer stage have been reported, only one clinical study by Karsten *et al*. demonstrated a significant relationship between high nestin levels and cancer stage.[24] Our aim here was to clarify the relationship between nestin and cancer stage by performing a meta-analysis of the literature in the field. This analysis provided evidence of an association between positive/high nestin and median/advanced cancer stage in different cancers, demonstrating a significant main effect of positive/high nestin versus negative/low nestin. Analyses of nestin association stratified according to lymph node involvement (N_1-2_ *vs*. N_0_) and cancer category were also statistically significant.

The relationships established here suggest that positive/high nestin can be used as an added factor in determining TNM stage and judging cancer malignancy, especially for lung cancer. Regarding to focus at the association of nestin with lung cancer staging, we have done some analysis on the relationship between nestin and T staging or N staging. Analyses of nestin association stratified according to T staging (T_3-4_ *vs.* T_1-2_) or N staging (N_1-2_ *vs*. N_0_) and lung cancer category were also statistically significant. This analysis provided evidence of an association between positive/high nestin and median/advanced cancer stage in lung cancer, demonstrating a significant main effect of positive/high nestin versus negative/low nestin. Further investigations of nestin and lung cancer stage on a larger scale are needed to verify this result and firmly establish nestin as a cancer malignancy biomarker.

Some limitations of this meta-analysis should be noted. Firstly, this meta-analysis was based on pooled positive or high nestin data; thus, we were only able to address the relationship between positive/high nestin and cancer stage, and not individual positive or high-nestin values. Secondly, a small-study effect, in which results reported in smaller studies have a disproportionate effect on the final analysis, could not be avoided because of the relatively small size of some of the studies. Ultimately, additional large scale-studies with more detailed individual data are warranted to further validate the relationship of nestin with cancer stage.

## CONCLUSIONS

In summary, our meta-analysis provides evidence of an association between positive/high nestin and cancer stage, suggesting that knowledge of nestin expression status could facilitate cancer staging, especially in lung cancer.

## MATERIALS AND METHODS

### Publication search

Relevant articles published as of June 2014 were retrieved from multiple databases, including PubMed, ISI Web of Knowledge, Embase, and the Chinese National Knowledge Infrastructure (CNKI) using the keywords nestin, malignancy, neoplasm, cancer staging and TNM staging. Articles with case-control or cohort studies related to the association of positive/high nestin and cancer staging were collected.

### Inclusion criteria

The following criteria were used to select publications for further meta-analysis: (1) published in English or Chinese, regardless of publication date; (2) evaluated associations between positive/high nestin and cancer stage; (3) confirmed cancer patients pathologically; (4) included detailed cancer/TNM staging data; and (5) contained at least two comparison groups (positive-nestin group *vs*. negative-nestin group, or high-nestin group *vs*. low-nestin group).

### Data collection

Publication details such as the first author's name, publication year, patients' country of origin and ethnicity, total number of patients, cancer type, median/mean age of the study population, and disease stage were collected for each eligible publication. Positive or high nestin and cancer/TNM stage were end points of interest.

### Statistical analysis

Patients were divided into positive/high nestin and negative/low nestin groups, and the associations between positive/high nestin and cancer stage were measured by determining odds ratios (ORs) with 95% conﬁdence intervals (CIs). The signiﬁcance of pooled ORs was determined by the Z test. Statistical heterogeneity among studies was assessed using Q and *I^2^* statistics, where variation due to heterogeneity or random error identified with Q tests were further defined according to *I^2^* values, as follows: *I^2^* = 0–25%, no heterogeneity; *I^2^* = 25–50%, moderate heterogeneity; *I^2^* = 50–75%: large heterogeneity; and *I^2^* = 75–100%, extreme heterogeneity.[26] A fixed-effects model was applied in the initial analysis, and if significant heterogeneity existed, a confirmed random-effects model was used. Publication bias was evaluated using funnel plots. All statistical analyses were carried out with Review Manager version 5.0 (Revman; The Cochrane Collaboration, Oxford, UK). All *P*-values in the meta-analysis were two-sided, and a *P*-value < 0.05 was considered significant.
